# Associated factors with neonatal near miss in twin pregnancies in a public referral maternity unit in Brazil

**DOI:** 10.4274/jtgga.galenos.2021.2020.0176

**Published:** 2021-02-24

**Authors:** Fernanda Nogueira Barbosa Lopes, Ana Paula Mendes Gouveia, Ocília Maria Costa Carvalho, Antônio Brazil Viana Júnior, Álvaro Jorge Madeiro Leite, Edward Araujo Júnior, Francisco Herlânio Costa Carvalho

**Affiliations:** 1 Department of Community Health, Federal University of Ceará, Fortaleza, Brazil; 2 Department of Maternal and Child Health, Federal University of Ceará, Fortaleza, Brazil; 3 Department of Obstetrics, Paulista School of Medicine, Federal University of São Paulo (EPM-UNIFESP), São Paulo, Brazil; 4 Medical course, Municipal University of São Caetano do Sul, São Paulo, Brazil

**Keywords:** Near miss healthcare, morbidity, twin pregnancy, perinatal care

## Abstract

**Objective:**

The aim was to analyze the factors associated with neonatal near miss (NNM) in twin pregnancies in a public referral maternity unit in Brazil.

**Material and Methods:**

This retrospective, cross-sectional study included 697 twin newborns. Cases of fetal and neonatal deaths were excluded. Neonates were divided into those meeting NNM criteria (5 min Apgar score <7, birth weight <1,500 g, gestational age at delivery <32 weeks, use of mechanical ventilation or congenital malformation, transfer before 28 days of life) and those who did not. In the bivariate analysis, the chi-square and Fisher’s exact tests were used. Variables with a p-value ≤0.20 were subjected to the multiple analyses, which followed the Poisson regression model.

**Results:**

The cohort consisted of 130 (18.7%) neonates meeting NNM criteria and 567 (81.3%) with no NNM criteria after multiple analyses, the following variables were associated with NNM: no previous pregnancy, prevalence ratio (PR): 1.38 [95% confidence interval (CI), 1.03-1.85]; >3 previous pregnancies, PR: 1.93 (95% CI, 1.38-2.69); premature rupture of membranes, PR: 1.50 (95% CI, 1.70-2.12); intrauterine growth restriction, PR: 2.28 (95% CI, 1.53-3.33); premature labor, PR: 1.63 (95% CI, 1.13-2.35); resuscitation in the delivery room, PR: 1.80 (95% CI, 1.24-2.62); and transfusion of blood products, PR: 4.44 (95% CI, 3.14-6.28).

**Conclusion:**

The study findings indicate that having had 0 or >3 previous pregnancies, premature rupture of the membranes, intrauterine growth restriction, resuscitation in the delivery room, premature labor, and transfusion of blood products were associated with NNM in twin pregnancies.

## Introduction

The incidence of twin pregnancies is increasing worldwide (1), with a mean incidence of 13.1/1,000 live births (LB) (2). In three decades, in the USA, the birth rate of twins has risen 76%, attributed to the increase in the average maternal age and the emergence of new assisted reproduction technologies (1,3). In Brazil, between 2011-2014, twin births represented 1.13% of LB, with 0.98% in the state of Ceará (4). Studies have shown an association between twinning and a five minute (5 min) Apgar score <7, low birth weight, neonatal intensive care unit admission, and a consequent increase in neonatal morbidity and mortality rates (5,6,7).

The concept of neonatal near miss (NNM) is used for obstetrical events that almost resulted in the death of newborns from 0 to 28 days of life (8). However, globally accepted criteria for identifying cases have not yet been defined, which is a challenge for the identification and real estimate of its impact (9). Such criteria will depend on the production of evidence to enable the identification of really serious cases, the possibility of easy data collection in terms of clinical care and the applicability to different scenarios (10,11).

Research of various concepts of NNM showed associations with twin pregnancy (12,13,14,15). This term refers to cases of newborns who almost died as a result of some serious complications (11). The identification of these cases and application of this concept to the neonatal population is an important tool in the identification of deficiencies in the health services provided to the mother-baby dyad (8,16).

The choice of researching twin pregnancies, regardless of their classification by chorionicity, started from the need to identify and recognize the reality of twin deliveries that occurred in our institution, taking into account the absence of studies of this population. Considering the importance and scarcity of NNM studies, especially in twin pregnancies, this study aimed to analyze the factors associated with NNM in twin pregnancies in a public, tertiary care, referral maternity unit for high-risk pregnancies in Brazil.

## Material and Methods

This retrospective cross-sectional study identified twins among all live newborns born at the maternity school between January 2016 and December 2018. NNM cases were those that met at least one of the criteria published by Da Silva et al. (13): birth weight <1,500 g, 5 min Apgar score <7, use of mechanical ventilation, gestational age at delivery <32 weeks, and presence of congenital malformations.

Exclusion criteria were: cases of early and late neonatal death; transfers (before 28 days of life); conjoined twins; abortion (gestational age <20 weeks, weight <500 g); delivery of the first twin outside the hospital environment; and patients for whom information was incomplete or missing from the medical records.

In the assessment of sociodemographic characteristics, pre-existing clinical conditions, prenatal care, complications during pregnancy, and childbirth, the pregnant women were the unit of analysis. They were classified as NNM (those for whom at least one twin met one or more NNM criteria) or non-NNM (those for whom neither twin met one or more NNM criteria). For variables related to the newborn’s health conditions, the neonates were the unit of analysis. For each mother, there were one or two newborns since one could be excluded from the analysis due to fetal or neonatal death; the woman and her surviving newborn could still be included.

A form containing questions directed to the following study variables was constructed for data collection: maternal sociodemographic characteristics, obstetric clinical history, conditions related to pregnancy, NNM criteria, maternal outcome, conditions related to delivery, conditions related to the newborn, criteria for NNM and neonatal outcome. This instrument was reviewed before the data collection started, a pilot test was carried out in order to identify flaws and test its applicability. The information was extracted from medical records and/or other medical records as a declaration of LB.

The following near miss indicators were also calculated and adapted to the neonatal context by Pileggi et al. (8) and Pileggi-Castro et al. (17): NNM rate, severe neonatal outcome rate, early neonatal mortality index, NNM/neonatal death ratio, and early neonatal mortality rate.

The Federal University of Ceará Local Ethic Committee approved the study under the certificate of presentation for ethical appraisal (approval number: 04091418.7.0000.5050). Consent was also obtained when the participants signed the Term of Faithful Depositary prior to the data collection.

### Statistical analysis

The data were analyzed using SPSS version 23.0 (SSP Inc., Chicago, IL, USA). For univariate analysis, chi-square and Fisher’s exact tests were used, when appropriate. Missing information was not used in the significance calculation. Variables with values of p≤0.20 were tested again using multiple analyses and a Poisson regression model with robust variance, avoiding possible confounding variables. The prevalence ratio (PR) and 95% confidence intervals (CI) were calculated. The variables with values of p<0.05 on the multiple analyses were included in the final regression model.

## Results

Between January 2016 and December 2018, a total of 14,870 births occurred at the surveyed institution and of these 904 (6%) were due to twin pregnancies. One hundred and 120 twins were excluded because they met the exclusion criteria. Of them, 87 (28 fetal deaths, 42 early neonatal deaths, and 17 late neonatal deaths) were excluded. In the population eligible for analysis, 567 (81.3%) live newborns met NNM criteria and 130 (18.7%) did not meet NNM criteria for a total of 697 twin studies.

Based on the proposed quality monitoring and neonatal care indicators (8), a NNM rate of 171.9/1,000 LB was obtained, an early neonatal mortality rate of 55.6/1,000 LB, index early neonatal mortality of 22.2%, severe neonatal outcome rate of 227.5/1,000 LB, and 2.2 cases of NNM for each neonatal death.

None of the variables related to maternal sociodemographic characteristics showed a statistically significant association with the NNM cases. Most of the women surveyed were 19-34 years of age (77.8%), were multiparous, and had no previous history of abortion. Obesity (17%) was the most prevalent pre-existing condition, followed by chronic arterial hypertension (9.3%) and syphilis (2.7%). Among the clinical conditions studied, diabetes mellitus (p=0.034), kidney disease (p=0.019), and thyroid disease (p=0.013) were significantly different between the two groups (Table 1).

Prenatal care, complications during pregnancy and childbirth data are shown in Table 2.

Preterm birth occurred in 69.4% and 66.9% of newborns weighed <2,500g. In addition to the variables used to identify cases of NNM, cesarean delivery, 1 min Apgar score >7, transfusion of blood products, resuscitation in the delivery room, and length of stay >28 days were also significantly associated with NNM (Table 3).

The variables used as a defining criterion for NNM outcome were removed from the multiple analyses. The variables number of previous pregnancies; premature rupture of membranes; intrauterine growth restriction; resuscitation in the delivery room; premature labor and transfusion of blood products, were associated with NNM and remained in the final model (Table 4).

## Discussion

The assessment of severe neonatal morbidity is a new health indicator contributing to the identification of factors in the health system requiring remedial action, assessment of care quality, and guidance for decision-making by health managers and providers (11). These measures may contribute to the reduction of neonatal mortality rates in addition to allowing the calculation of ratio/rates between deaths and cases of near miss, better specifying health care indicators for the most severe cases (8,17).

According to the classification criteria applied (13), 2.2 cases of near miss were identified for each neonatal death, which was higher than the findings in earlier Brazilian studies that addressed the feasibility of the NNM concept (13,14). The scarcity of research of NNM in twins makes it difficult to compare these rates and rates within this specific population, yet our findings suggest that twins have worse outcomes for severe neonatal morbidity.

Variables, such as low maternal education level, race/skin color of mixed mothers, women without partners, and lower socioeconomic classes are widely debated since they show an association with increased neonatal morbidity and mortality (14,18). Although twin pregnancies are associated with Brazilian regions with the highest human development index, higher education level, and high maternal age (>35 years), this study showed no relationship between NNM and maternal socioeconomic and demographic conditions (4). In line with this finding, two studies in Brazilian maternity hospitals also reported no such association (15,19). It should be noted that the surveyed population included only twin pregnancies born in a public maternity hospital.

Prematurity occurs in about 50% of Brazilian twin deliveries and is almost 5.0 times more prevalent when compared to singleton pregnancies, being up to 12 times higher in extremely preterm infants. Premature labor is also associated with a 5 min Apgar <7. In premature infants aged 32-36 weeks the risk of Apgar <7 at the 5^th^ min is 2.5 and in newborns <32 weeks this risk may be 30 times greater, which may be related to adverse neonatal outcomes (4). The rate of prematurity (69.4%) was higher than that reported in previous Brazilian studies, a fact that can be attributed to the research being performed exclusively in a reference maternity hospital for high-risk pregnancies. Other studies also reported an association between NNM and preterm birth, 1 min Apgar score <7, premature rupture of membranes, and neonatal resuscitation, as was found in the current cohort (13,14).

The accuracy of the first minute Apgar score has been investigated as a diagnostic test or marker for the presence of asphyxia and indicates that less than half of newborns with low Apgar scores are asphyxiated, according to the gasometric criteria (20). The purpose of this study was not to analyze hypoxemic events, as the 5 min Apgar score <7 was used as an NNM defining criterion, as it is widely used as marker of neonatal morbidity.

In a Brazilian study, pregnant women with inadequate prenatal care were more susceptible to having spontaneous premature labors. The prenatal coverage in Brazil has advanced in the last 15 years. However, access failure, late start, and incomplete execution of procedures still occur. Data reflect gaps in assistance, in addition to the historical situation of regional and socioeconomic inequality that is present in the country (21,22). Lima et al. (19) found an association between <6 prenatal consultations and NNM, which resulted in a four times greater risk of NNM. In our findings, 99.2% of women attended at least one consultation and 70.1% attended >6 consultations, showing increased access, but this did not guarantee better care quality, especially in higher-risk pregnancies. Assessment of the number of consultations alone does not result in better assistance. Assessing the quality of the provided services was not the objective of this study.

This series revealed no relationship between birth order and NNM. Much has been discussed about the influence of birth order on worse neonatal outcomes, specifically that the second twin has worse perinatal outcomes (4). Other authors have demonstrated that if the birth conditions between the first and second twins are uniform, the birth order will not influence perinatal outcomes (23).

Monochorionic pregnancies are generally associated with a higher risk of perinatal complications and perinatal morbidity and mortality compared to dichorionic pregnancies, since they have specific obstetric complications, such as twin-to-twin transfusion syndrome (TTTS), twin anemia-polycythemia sequence (TAPS), and twin reversed arterial perfusion (TRAP) sequence (6,24). Studies have shown that monochorionic twins have a higher incidence of prematurity, premature labor, olygohydramnios/polyhydramnios, intrauterine growth restriction, lower maternal age, use of mechanical ventilation, lower gestational age at delivery, low birth weight, and seven times greater chance of perinatal mortality (25,26,27). In this study, no significant association was observed between chorionicity and NNM, thus diverging from the results mentioned above. More recent studies have suggested a downward trend in perinatal morbidity and mortality in monochorionic pregnancies when there is an early diagnosis and intensive surveillance during prenatal care (28,29,30,31,32). The research institution in the present study, a maternity school, has a fetal medicine service, and the professionals who work there have expertise in handling high-risk pregnancies. We suggest that this may have resulted in their being no effect of chorionicity in our cohort.

Cesarean delivery plays an important role in reducing perinatal risks and, consequently, increasing newborn survival (30). Although mode of delivery did not appear in the explanatory model of factors associated with NNM, the significant association of NNM with vaginal delivery found here can be explained by the fact that twin pregnancies are associated with several maternal and fetal complications that require therapeutic cesarean section or a greater risk of neonatal death in cases of vaginal delivery (18,31).

Among the predictor variables that remained in the final model, blood product transfusion appeared with a PR of 4.44 (95% CI, 3.14-6.28) and a neonatal resuscitation PR of 1.80 (95% CI, 1.24-2.62). These two variables were also identified in a study that investigated the same outcome and made up some of the management criteria studied by the Latin American Center for Perinatology, because of their association with NNM (19). Efforts should be concentrated to avoid preventable complications of twin pregnancy, avoiding, for example, premature labor to achieve better neonatal outcomes since premature birth directly influences neonatal morbidity (32).

To verify the correlation between advanced maternal age and parity with NNM, Martinelli et al. (12) found an association between advanced maternal age and NNM in nulliparous women (odds ratio: 1.62; 95% CI, 1.05-2.50) and multiparous women (odds ratio: 1.51; 95% CI, 1.20-1.91) when women aged 20-29 years were compared. Although the research above included singleton and multiple pregnancies, the data corroborated the current findings.

The present study included a considerable sample and is a pilot in this institution, examining twin pregnancies with NNM outcomes. However, its limitations need to be recognized. Some complications related to monochorionic pregnancies (TTTS and TAPS) were not properly assessed and therefore were not presented here. The absence of a detailed description of ultrasound examinations hindered this analysis. There was no definition of chorionicity, even after macroscopic analysis of the placenta, in 4.4% of cases. In other situations, the lack of data in the medical records prevented the collection of some variables, such as body mass index in 31.2% of patients. These data can be extrapolated to aproximate the reality in many Brazilian and Latin American maternity hospitals: a tertiary-level institution that serves people of low socioeconomic level and does not feature highly complex technology such as equipment used to perform fetoscopy and laser therapy.

The results presented here show that it is necessary to target health policies, especially actions aimed at the socially vulnerable in the population and in conditions of high-risk pregnancies. There is also a need for studies that can compare NNM outcomes in twin versus single pregnancies, including research into management criteria recently listed by the Latin American Center for Perinatology to better investigate the association of these factors in twin pregnancies. This direct comparison was not made in this study. It is also necessary to reassess these results in the long term, especially after offering the best technological equipment. The use of protocols that identify neonatal morbidity criteria can help better guide twin care.

## Conclusion

These findings allow us to understand that risk of NNM in twinning is associated with the number of previous pregnancies, premature labor, premature rupture of membranes, intrauterine growth restriction, need for resuscitation in the delivery room, and transfusion of blood products. In clinical practice, these results can assist with the implementation of protocols and measures to identify high-risk situations during obstetric and neonatal care and improve neonatal results. Future studies into the topic are essential, especially to better assess conditions related to chorionicity and outcomes between first and second twins.

## Figures and Tables

**Table 1:**
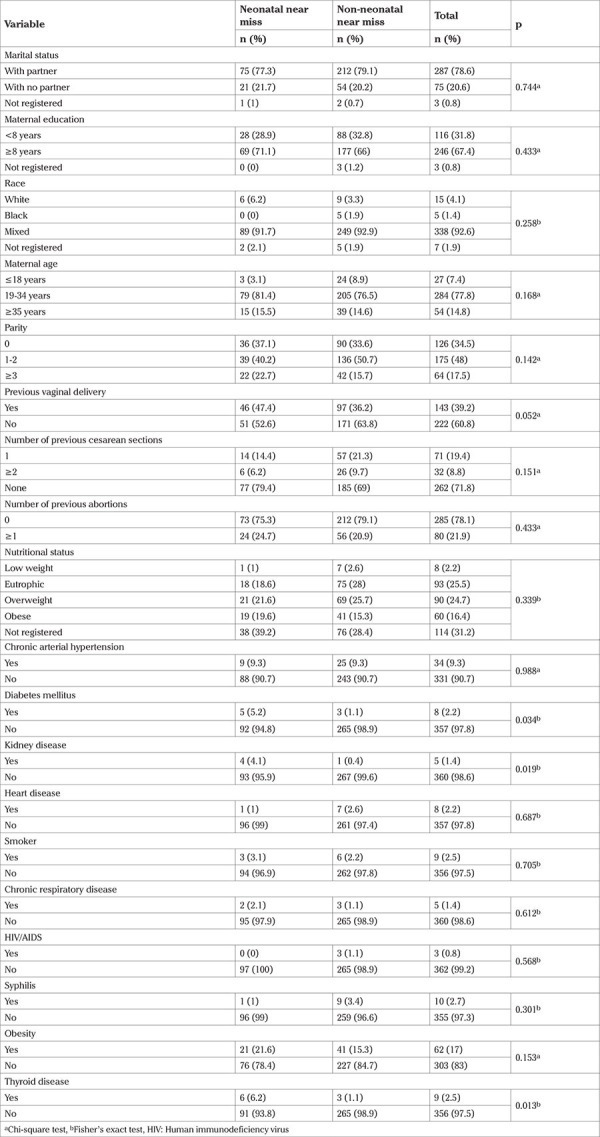
Sociodemographic characteristics and pre-existing clinical conditions of mothers of twins considered neonatal near miss and non-neonatal near miss.

**Table 2:**
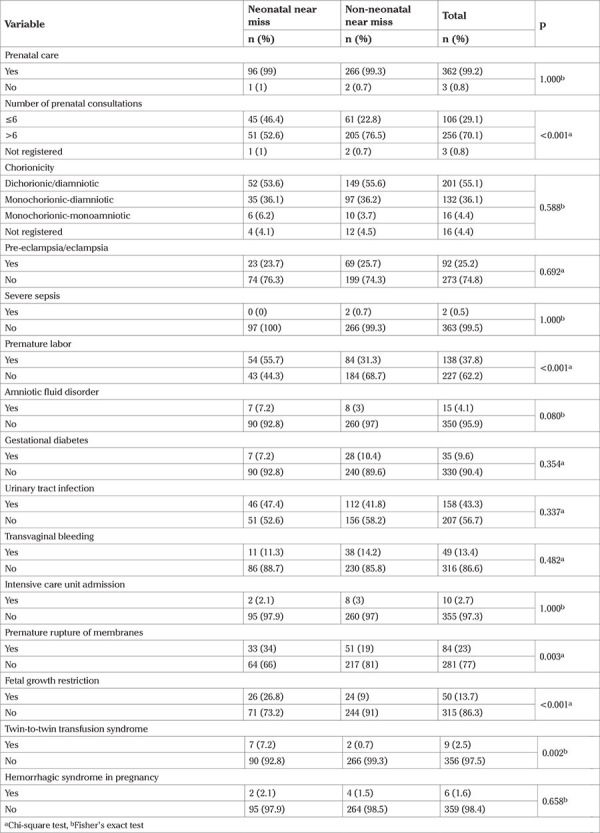
Characteristics of prenatal care, complications during pregnancy, and delivery in mothers of twins considered neonatal near miss versus non-neonatal near miss

**Table 3:**
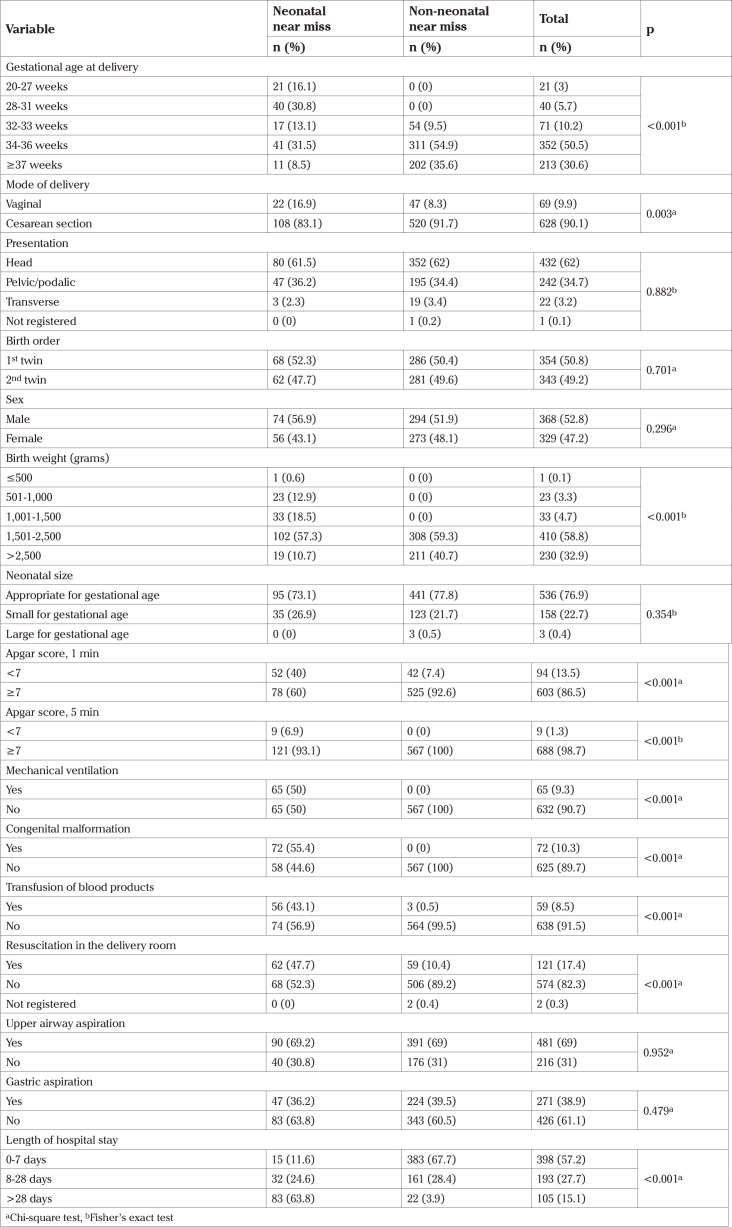
Birth conditions, newborn health, and neonatal care among twins considered neonatal near miss and non-neonatal near miss

**Table 4:**
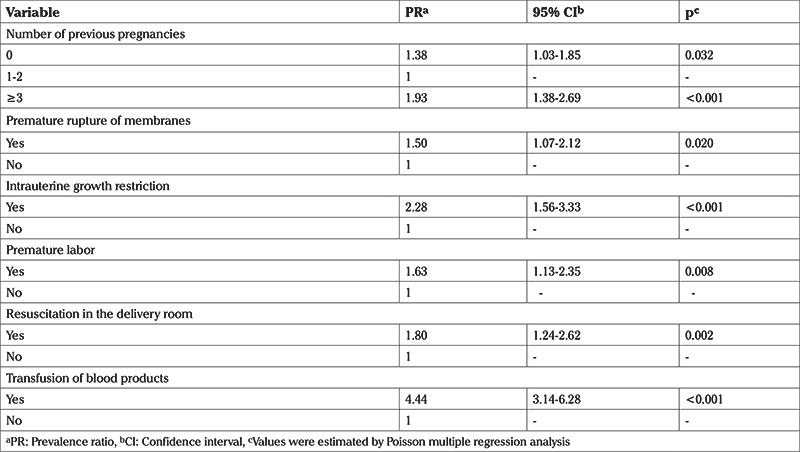
Final model of factors associated with neonatal near miss in twin pregnancies
